# Evaluation of Socially-Aware Robot Navigation

**DOI:** 10.3389/frobt.2021.721317

**Published:** 2022-01-12

**Authors:** Yuxiang Gao, Chien-Ming Huang

**Affiliations:** Department of Computer Science, The Johns Hopkins University, Baltimore, MD, United States

**Keywords:** socially-aware navigation, human-robot interaction, mobile robots, robot navigation, human-aware navigation

## Abstract

As mobile robots are increasingly introduced into our daily lives, it grows ever more imperative that these robots navigate with and among people in a safe and socially acceptable manner, particularly in shared spaces. While research on enabling socially-aware robot navigation has expanded over the years, there are no agreed-upon evaluation protocols or benchmarks to allow for the systematic development and evaluation of socially-aware navigation. As an effort to aid more productive development and progress comparisons, in this paper we review the evaluation methods, scenarios, datasets, and metrics commonly used in previous socially-aware navigation research, discuss the limitations of existing evaluation protocols, and highlight research opportunities for advancing socially-aware robot navigation.

## 1 Introduction

Fueled by advances in artificial intelligence (AI) technologies, mobile robots are realizing increased adoption in various delivery-based industries, from mail^1^ and packages^2^ to pizza.^3^ Mobile robots designed for these consumer-facing services must not only navigate safely and efficiently to their destinations but also abide by social expectations as they move through human environments. For example, it is desirable for mobile robots to respect personal space (Althaus et al., 2004), avoid cutting through social groups (Katyal et al., 2021), move at a velocity that does not distress nearby pedestrians (Kato et al., 2015), and approach people from visible directions (Huang et al., 2014) while maintaining relevant social dynamics (Truong and Ngo, 2018). Research that investigates robot capabilities for navigating in human environments in an efficient, safe, and socially acceptable manner is commonly recognized as socially-aware navigation—also known as human-aware navigation (e.g., Kruse et al., 2013), socially compliant navigation (e.g., Kretzschmar et al., 2016), socially acceptable navigation (e.g., Shiomi et al., 2014), or socially competent navigation (e.g., Mavrogiannis et al., 2017).

While research on socially-aware navigation has expanded over the years (Kruse et al., 2013; Rios-Martinez et al., 2015; Charalampous et al., 2017; Pandey, 2017), there are no standard evaluation protocols—including methods, scenarios, datasets, and metrics—to benchmark research progress. Prior works on socially-aware robot navigation utilize a variety of evaluation protocols in custom settings, rendering comparisons of research results difficult. We argue that commonly agreed-upon evaluation protocols are key to fruitful progress, as observed in other research fields (e.g., computer vision). As an effort to productively advance socially-aware navigation, in this paper we review commonly used evaluation methods, scenarios, datasets, and metrics in relevant prior research. We note that our review focuses on evaluation protocols rather than the algorithmic methods and systems that enable socially-aware navigation. We further note that socially-aware navigation is strongly related to an array of research topics, including human trajectory prediction, agent and crowd simulation, and robot navigation; some of the evaluation protocols reviewed in this paper may apply to these related research areas. Our review complements the recommendation for evaluation of embodied navigation suggested by Anderson et al. (2018) and can be consulted along with other general evaluation guidelines for human-robot interactions (Steinfeld et al., 2006; Young et al., 2011; Murphy and Schreckenghost, 2013).

The reminder of this paper is organized as follows. In Section 3, we present evaluation methods, scenarios, and datasets commonly used for evaluating socially-aware navigation. In Section 4, we review evaluation metrics and focus on the aspects of navigation performance, behavioral naturalness, human discomfort, and socialbility. We conclude this review with a discussion of limitations of existing evaluation protocols and opportunities for future research.

## 2 Methodology

**FIGURE 1:**
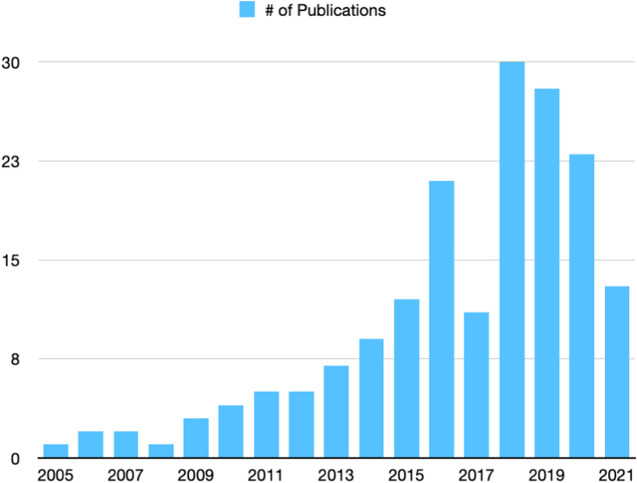
Number of publications collected by year.

**FIGURE 2:**
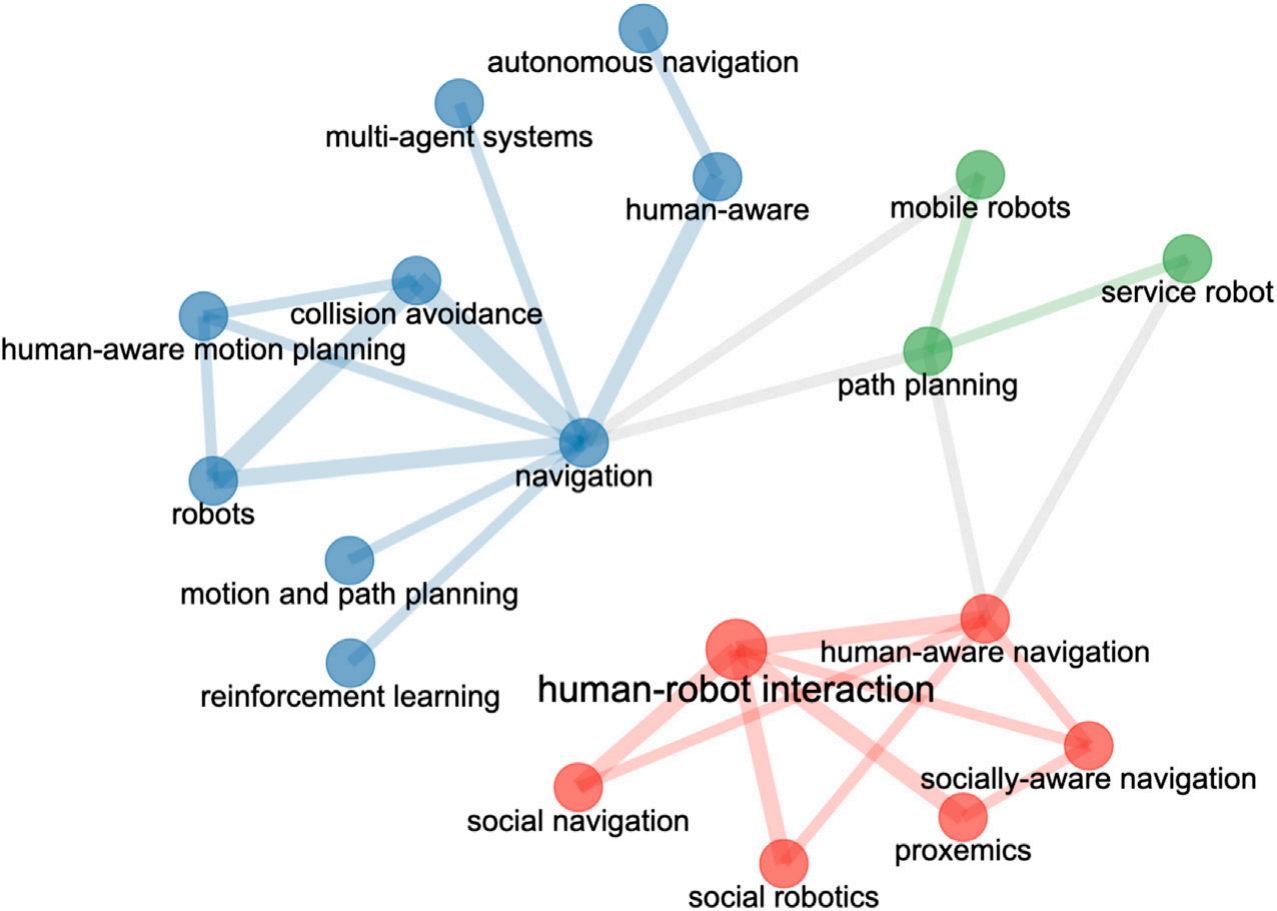
Co-occurrence network of the keywords appeared in the collected publications. The keywords are clustered using Louvain algorithm. This graph is generated using Bibilometrix (Aria and Cuccurullo, 2017), a bibliometrics analysis tool.

Methodologically, this paper can be considered as a literature review—“*a literature review reviews published literature, implying that included materials possess some degree of permanence and, possibly, have been subject to a peer-review process. Generally, a literature review involves some process for identifying materials for potential inclusion—whether or not requiring a formal literature search—for selecting included materials, for synthesizing them in textual, tabular or graphical form and for making some analysis of their contribution or value*” (Grant and Booth, 2009). We focus on reviewing evaluation protocols for socially-aware robot navigation. While we did not follow the scoping process used for a systematic review, we identified materials (papers and datasets) for inclusion based on their relevance to the topic of socially-aware robot navigation and its evaluation methods. Specifically, we used keywords “socially-aware navigation,”“socially-acceptable navigation,” “human-aware navigation,” or “crowd-aware navigation” when searching papers through ACM Digital Library, IEEE Xplore, and ScienceDirect. We additionally included some preprints from ArXiv through Google Scholar searches. This process yielded 188 papers in our initial search. Upon further reviewing the titles and abstracts of the papers, we removed 11 papers that did not address socially-aware robot navigation. The remaining 177 papers were published between 2005 and 2021 (Figure 1). A co-occurrence network of the keywords of the included papers is shown in Figure 2; the network illustrates three clusters that approximately represent topics related to human-robot interaction or social aspects of navigation (red), algorithmic methods for navigation (blue), and navigation systems (green). The co-occurrence network was automatically generated through Bibilometrix (Aria and Cuccurullo, 2017), a bibliometrics analysis tool, using Louvain algorithm. Table 1 lists major venues where the 177 papers were published.

**TABLE 1:** Publication venues of the included 177 publications. Only venues that have more than five papers are listed.

Publication venues	#Of papers collected
**IEEE/RSJ International Conference on Intelligent Robots and Systems (IROS)**	21
**IEEE International Conference on Robot and Human Interactive Communication (RO-MAN)**	13
**IEEE International Conference on Robotics and Automation (ICRA)**	12
**International Journal of Social Robotics**	8
**IEEE Robotics and Automation Letters (RA-L)**	6
**ACM/IEEE International Conference on Human-Robot Interaction (HRI)**	5
**Others**	112

Upon collecting the 177 papers, we further reviewed the evaluation section of each paper and chose the studies that are representatives of the evaluation metrics, evaluation methods, datasets, and test scenarios described in the next section. Through this process, we observed that many of the evaluation metrics were originated from related works on neighboring research topics such as human trajectory prediction, autonomous robot navigation, and crowd simulation. As a result, we include relevant works on these topics to better understand the development of the evaluation methods in our report and discussion below.

## 3 Evaluation Methods, Scenarios, and Datasets

In this section, we describe evaluation methods, scenarios, and datasets commonly used in socially-aware navigation research, some of which apply directly to the problems of human trajectory prediction, crowd simulation, and general robot navigation.

### 3.1 Evaluation Methods

Mavrogiannis et al. (2019) classified the evaluation methods into three categories: simulation study, experimental demonstration, and experimental study. In this review, we follow a similar but more granular classification based on the type, location, and goal of the evaluation methods. Specifically, we focus on four evaluation methods—case study, simulation and demonstration, laboratory study, and field study—regularly used in socially-aware navigation research. Each method has its own advantages and disadvantages and is often used at different stages of development.

#### 3.1.1 Case Studies

Because navigating among people in human environments involves complex, rich interactions, it is common to break down socially-aware navigation into sets of primitive, routine navigational interactions such as passing and crossing (Table 2). As such, prior research has utilized case studies to illustrate robot capabilities in handling these common navigational interactions. Said case studies usually involve prescribed interaction behaviors (e.g., asking the test subjects to walk in a predetermined direction or behave as if they were walking together) and environmental configurations. For example, Pacchierotti et al. (2006) studied how a person and a robot may pass each other in a hallway environment; their study involved different human behaviors, such as moving at a constant speed or stopping in the middle of the hallway, and illustrated how the robot may respond to those behaviors. Similarly, Kretzschmar et al. (2016) reported a study demonstrating how their inverse reinforcement learning approach allowed a robotic wheelchair to pass two people walking together in a hallway without cutting through the group. Truong and Ngo (2017) presented an illustrative study comparing their proactive social motion model (PSMM) against the social force model (SFM) in four experimental settings and showed that their model yielded a more socially acceptable navigation scheme. Case studies can also be presented *via* simulation; Rios-martinez et al. (2013) used a set of predefined simulated configurations of human behaviors (e.g., moving around and interacting with each other) to illustrate their proposed method for reducing discomfort caused by robot movements.

**TABLE 2:** Scenarios commonly used in evaluating socially-aware navigation. The publications that employ each scenario in simulation or real-world settings are listed respectively.

Interaction type	Illustrations	Used in simulation	Used in real-world settings
**Passing**	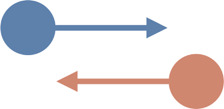	Chen et al. (2017a); Vega et al. (2019b); Yang and Peters (2019); Randhavane et al. (2019); Pandey and Alami (2010); Pérez-D’Arpino et al. (2020)	Butler and Agah (2001); Pacchierotti et al. (2006); Kretzschmar et al. (2016); Okal and Arras (2016);
**Crossing**	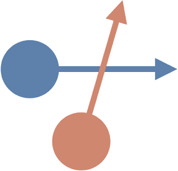	Alahi et al. (2016); Chen et al. (2017a); Chen K. et al. (2019); Sui et al. (2019); Manso et al. (2019); Khambhaita and Alami (2020); Nishimura and Yonetani (2020); Daza et al. (2021)	Guzzi et al. (2013b); Kretzschmar et al. (2016); Johnson and Kuipers (2019); Mavrogiannis et al. (2019)
**Overtaking**	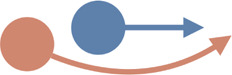	Kirby (2010); Pandey and Alami (2010); Anvari et al. (2015); Chen et al. (2017a)	Pandey and Alami (2010); Šochman and Hogg (2011); Robicquet et al. (2016); Yang and Peters (2019)
**Approaching**	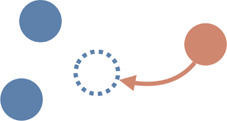	Turner (1981); Sisbot et al. (2005); Truong and Ngo (2018); Johnson and Kuipers (2019); Truong and Ngo (2019)	Butler and Agah (2001); Satake et al. (2009); Kato et al. (2015); Truong and Ngo (2018); Joosse et al. (2021)
**Following, leading, and accompanying**	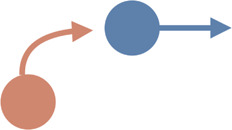	Ferrer et al. (2013b); Yao et al. (2019)	Ferrer et al. (2013a); Ferrer et al. (2013b); Ferrer et al. (2017); Du et al. (2019); Repiso et al. (2020)
**Combined**	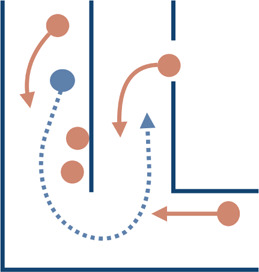	Okal and Arras (2014); Okal and Arras (2016); Pandey (2017); Yang and Peters (2019)	Shiomi et al. (2014); Truong and Ngo (2018); Vega et al. (2019b)

#### 3.1.2 Simulation and Demonstrations

Simulation experiments have been regularly utilized in recent years due to advances in reinforcement learning and data-driven approaches to socially-aware navigation (e.g., Chen C. et al., 2019; Li et al., 2019; Liu Y. et al., 2020). They are particularly useful for agile development and systematic benchmarking. Simulation experiments are typically supplemented by physical demonstrations to exhibit intended robot capabilities; the objective of these demonstrations is to illustrate that the proposed algorithmic methods work not only in simulated setups but also in the physical world with a real robot. For instance, Chen et al. (2020) first evaluated their method for crowd navigation in a simulated circle crossing scenario with five agents, after which they provided a demonstration of their method using a Pioneer robot interacting with human subjects. Katyal et al. (2020) and Liu L. et al. (2020) followed a similar method, including a simulation evaluation and a physical demonstration in their investigation of adaptive crowd navigation. Prior works that report this type of physical demonstration typically provide supplementary videos of the demonstrations (e.g., Jin et al., 2019).

Because of the popularity of simulation-based evaluation, an array of simulation platforms have been developed for robot navigation, ranging from simplistic 2D simulation [e.g., Stage (Gerkey et al., 2003) and CrowdNav (Chen C. et al., 2019), pedsimROS (Okal and Linder, 2013), MengeROS (Aroor et al., 2017)], to high-fidelity simulation leveraging existing physics and rendering engines [e.g., Webots,^4^ Gibson (Xia et al., 2018), and AI2-THOR (Kolve et al., 2019)] and virtualized real environments [e.g., Matterport3D (Chang et al., 2017)]. Among these efforts, the following simulation platforms address socially-aware navigation specifically:

• *PedsimROS* (Okal and Linder, 2013) is a 2D simulator based on *Social Force Model* (SFM) (Helbing and Molnár, 1995). It is integrated with the ROS navigation stack and enables easy simulation of large crowds in real time.
• *MengeROS* (Aroor et al., 2017) is a 2D simulator for realistic crowd and robot simulation. It employs several backend algorithms for crowd simulation, such as *Optimal Reciprocal Collision Avoidance* (ORCA) (Van Den Berg et al., 2011), *Social Force Model* (SFM) (Helbing and Molnár, 1995), and PedVO (Curtis and Manocha, 2014).
• *CrowdNav* (Chen C. et al., 2019) is a 2D crowd and robot simulator that serves as a wrapper of OpenAI Gym (Brockman et al., 2016), which enables training and benchmarking of many reinforcement learning based algorithms.
• *SEAN-EP* (Tsoi et al., 2020) is an experimental platform for collecting human feedback on socially-aware navigation in online interactive simulations. In this web-based simulation environment, users can control a human avatar and interact with virtual robots. The platform allows for easy specification of navigation tasks and the distribution of questionnaires; it also supports simultaneous data collection from multiple participants and offloads the heavy computation of realistic simulation to cloud servers. Its web-based platform makes large-scale data collection from a diverse group of people possible.
• *SocNavBench* (Biswas et al., 2021) is another benchmark framework that aims to evaluate different socially-aware navigation methods with consistency and interpretability. As opposed to most simulation-based approaches where agent behaviors are generated from crowd simulation [e.g., using Optimal Reciprocal Collision Avoidance (ORCA) (Van Den Berg et al., 2011) or Social Force Model (SFM) (Helbing and Molnár, 1995)], human behaviors in SocNavBench are grounded in real-world datasets (i.e., UCY and ETH datasets) (Section 3.3). SocNavBench renders photorealistic scenes based on the trajectories recorded in these datasets and employs a set of evaluation metrics to measure path (e.g., path irregularity) and motion (e.g., average speed and energy) quality and safety (e.g., closest collision distance).
• The *CrowdBot* simulator (Grzeskowiak et al., 2021) is another benchmarking tool for socially-aware navigation that leverages the physics engine and rendering capabilities of Unity and the optimization-based Unified Microscopic Agent Navigation Simulator (UMANS) (van Toll et al., 2020) to drive the behaviors of pedestrians.

In addition to shared platforms for simulation-based evaluation, several online technical competitions have sought to benchmark socially-aware navigation. For instance, the *TrajNet++ Challenge* ^5^ focuses on trajectory prediction for crowded scenes and the *iGibson Challenge* ^6^ includes a social navigation task contextualized in indoor navigational interactions with human avatars.

#### 3.1.3 Laboratory Studies

As opposed to case studies, which often involve prescribing human test subjects’ behaviors (e.g., having them intentionally walk toward the test robot), laboratory studies utilize experimental tasks to stimulate people’s natural behaviors and responses within specific contexts. Laboratory studies can be either controlled experiments or exploratory studies. Controlled experiments allow for statistical comparisons of navigation algorithms running on physical robots in semi-realistic environments; we note that controlled laboratory experiments contrast with simulation experiments, which lack the fidelity to represent real-world human-robot interactions. As an example, Mavrogiannis et al. (2019) designed an experimental task allowing three participants and a robot to move freely between six stations following a specified task procedure. A total of 105 participants were recruited for this experiment and a variety of objective and subjective metrics were collected to assess and compare three navigation strategies: Optimal Reciprocal Collision Avoidance (ORCA), Social Momentum (SM), and tele-operation. Additionally, Huang et al. (2014) evaluated how a humanoid robot may signal different levels of friendliness toward participants *via* movement behaviors—such as approach speed and direction of approach—in a mock museum setup.

Laboratory studies may also be exploratory, allowing researchers to gain early, prompt feedback from users without controlled experimentation. For instance, Bera et al. (2019) conducted an exploratory in-person lab study with 11 participants to investigate their perceptions of a robot’s navigational behaviors in response to their assumed emotions.

#### 3.1.4 Field Studies

While laboratory experiments allow for controlled comparisons, they bear reduced ecological validity; to address this limitation, field studies are used to explore people’s interactions with robots in naturalistic environments. The pioneering tour guide robots RHINO (Burgard et al., 1998) and MINERVA (Thrun et al., 1999) were deployed in museums to study their collision avoidance behaviors and how people reacted to them. More recently, Satake et al. (2009) conducted a field deployment in which a mobile robot approached customers in a shopping mall to recommend shops; they explored different approach strategies and examined failed attempts. Similarly, Shiomi et al. (2014) investigated socially acceptable collision avoidance and tested their methods on a mobile robot deployed in a shopping mall for several hours with the objective of interacting with uninstructed pedestrians. Trautman et al. (2015) collected 488 runs of their experiment in a crowded cafeteria across 3 months to validate their algorithm. A benefit of deploying robots in the field is that they may reveal unexpected human behaviors; for instance, it was observed that young children “bully” a deployed mobile robot (e.g., intentionally blocking its way), which subsequently led to new research on how to recognize and avoid potential bullying behaviors in the field (Brščić et al., 2015). All in all, field studies are difficult to execute due to the unstructured, complex nature of real-world interactions—but are vital in evaluating socially-aware navigation and may offer insights that are otherwise impossible to discover in laboratory studies.

### 3.2 Primitive Scenarios

In this section, we describe common primitive scenarios found in the evaluation methods discussed in the previous section. Table 2 summarizes primitive scenarios in evaluating socially-aware navigation by the nature of the interactions involved. These scenarios include:

• Passing: This scenario captures interactions in which two agents or groups are heading in opposite directions, usually in constrained spaces such as hallways or corridors, and need to change their respective courses to pass each other.
• Crossing: This scenario captures interactions in which two agents or groups cross paths in an open space; it also considers if one of the agents or groups is stationary. Common examples of this scenario are circle crossing, where all agents are initiated on points of a circle (e.g., Chen C. et al., 2019; Nishimura and Yonetani, 2020), and square crossing, where all agents are initiated on the corners of a square (e.g., Guzzi et al., 2013b).
• Overtaking: This scenario captures interactions in which two agents or groups are heading in the same direction and one of them overtakes or passes the other.
• Approaching: This scenario captures interactions in which a robot intends to approach or join a stationary or moving group or individual. This scenario is observed when a robot attempts to join a static conversational group (e.g., Truong and Ngo, 2018; Yang et al., 2020), initiate an interaction (e.g., Kato et al., 2015) or follow a moving social group (e.g., Yao et al., 2019).
• Following, leading, and accompanying: This scenario captures interactions in which a robot intends to join a moving group by following (e.g., Yao et al., 2019), leading (e.g., Chuang et al., 2018), or accompanying the group side-by-side (e.g., Ferrer et al., 2017; Repiso et al., 2020).

### 3.3 Datasets

Table 3 details a number of datasets of human movement that are regularly used in developing algorithms for and evaluating socially-aware navigation systems. These datasets typically capture human movement in terms of trajectories or visual bounding boxes in various indoor and outdoor environments.

**TABLE 3:** Datasets used in socially-aware navigation.

Name	Year	# Of people	# Of scenes	Scene type	View type	Sensor type	Annotations	Publications
**UCY** (Lerner et al., 2007)	**2007**	**786**	**3**	**Outdoor**	**Top-down**	**Mono**	**Trajectories, Gaze**	Lerner et al. (2007); Alahi et al. (2016); Robicquet et al. (2016); Charalampous et al. (2016); Gupta et al. (2018); Vemula et al. (2018); Amirian et al. (2019); Yao et al. (2019); Kothari et al. (2020); Liu Y. et al. (2020); Biswas et al. (2021)
**ETH** (Pellegrini et al., 2009)	**2009**	**750**	**2**	**Outdoor**	**Top-down**	**Mono**	**Trajectories, Group Membership**	Pellegrini et al. (2009); Alahi et al. (2016); Charalampous et al. (2016); Robicquet et al. (2016); Gupta et al. (2018); Vemula et al. (2018); Amirian et al. (2019); Yao et al. (2019); Liu Y. et al. (2020); Kothari et al. (2020); Biswas et al. (2021)
**Edinburgh Informatics Forum Pedestrian Database (EIPD)** (Majecka, 2009)	**2009**	**95 ,998**	**1**	**Outdoor**	**Top-down**	**Mono**	**Trajectories**	Majecka (2009); Luber et al. (2012); Rudenko et al. (2017)
**PETS2010**	**2010**	**—**	**8**	**Outdoor**	**Surveillance**	**Mono**	**—**	Bandini et al. (2014); Bastani et al. (2015); Ristani and Tomasi (2015)
**VIRAT** (Oh et al., 2011)	**2011**	**4,021**	**11**	**Outdoor**	**Surveillance**	**Mono**	**Trajectories**	Oh et al. (2011); Vasquez (2016)
**Town Centre** (Benfold and Reid, 2011)	**2011**	**230**	**1**	**Outdoor**	**Surveillance**	**Mono**	**Bounding Boxes**	Benfold and Reid (2011); Ristani and Tomasi (2015); Le and Choi (2018)
**Grand Central Station** (Zhou et al., 2012)	**2012**	**12 ,600**	**1**	**Indoor**	**Surveillance**	**Mono**	**Trajectories**	Zhou et al. (2012); Gaydashenko et al. (2018)
**CFF** (Alahi et al., 2014)	**2014**	**42 million**	**1**	**Outdoor**	**Top-down**	**RGB-D**	**Trajectories, Bounding Boxes**	Alahi et al. (2014); Liu et al. (2020b); Kothari et al. (2020)
**Stanford Drone Dataset** (Robicquet et al., 2016)	**2016**	**11 ,216**	**8**	**Outdoor**	**Top-down**	**Mono**	**Trajectories**	Robicquet et al. (2016); Sadeghian et al. (2018a); Sadeghian et al. (2018b); Amirian et al. (2019); Li et al. (2020)
**EgoMotion** (Park et al., 2016)	**2016**	**—**	**26**	**Indoor**	**FPV**	**RGB-D**	**Bounding Boxes**	Park et al. (2016)
**L-CAS** (Yan et al., 2017)	**2017**	**6,140**	**1**	**Indoor**	**FPV**	**RGB-D**	**Trajectories**	Yan et al. (2017); Kothari et al. (2020); Liu Y. et al. (2020)
**STRAND** (Hawes et al., 2017)	**2017**	**—**	**1**	**Indoor**	**FPV**	**RGB-D**	**—**	Hawes et al. (2017)
**WildTrack** (Chavdarova et al., 2018)	**2018**	**9,518**	**7**	**Outdoor**	**Surveillance**	**Mono**	**Trajectories, Bounding Boxes**	Chavdarova et al. (2018); Liu Y. et al. (2020); Kothari et al. (2020)
**JackRabbot Dataset** (Martín-Martín et al., 2021)	**2019**	**260**	**—**	**Both**	**FPV**	**RGB-D**	**Trajectories, Bounding Boxes**	Martín-Martín et al. (2021)

The datasets are used to train models for predicting pedestrian trajectories and for generating robot movement in the presence of pedestrians. In particular, they are commonly utilized in modern data-driven approaches to socially-aware navigation, such as deep learning methods (e.g., Alahi et al., 2016; Zhou et al., 2021; Kothari et al., 2020), reinforcement learning (e.g., Chen et al., 2017a; Li et al., 2019), and generative adversarial networks (GAN) (e.g., Gupta et al., 2018; Sadeghian et al., 2018a).

Datasets are also used to evaluate and benchmark the performance of socially-aware navigation (e.g., Biswas et al., 2021; Xia et al., 2018); for example, datasets ETH (Pellegrini et al., 2009) and UCY (Lerner et al., 2007) have been widely utilized in comparing navigation baselines (e.g., Sadeghian et al., 2018a; Bisagno et al., 2019; Gupta et al., 2018). One way to use the data of human trajectories in evaluation is to replace one of the human agents with the test robot agent and compare the robot’s trajectory with the corresponding prerecorded human trajectory; various evaluation metrics described in the next section may be used to quantify the differences.

## 4 Evaluation Metrics

In this section, we review common metrics used to evaluate socially-aware navigation. We begin by presenting metrics for assessing navigation performance in the presence of humans. We then review metrics for representing various aspects of social compliance; in particular, we focus on the three key aspects of social compliance in socially-aware navigation as proposed by Kruse et al. (2013): naturalness—capturing motion-level similarity between robots and people; discomfort—representing the level of annoyance, stress, or danger as induced by the presence of the robot; and sociability—encapsulating how well the robot follows the social norms expected by surrounding pedestrians.

### 4.1 Navigation Performance

In general, prior works used navigation efficiency (Guzzi et al., 2013a; Guzzi et al., 2013b; Mavrogiannis et al., 2018; Liang et al., 2020) and *success rate* (Burgard et al., 1998; Guzzi et al., 2013b; Jin et al., 2019; Liang et al., 2020; Nishimura and Yonetani, 2020; Tsai and Oh, 2020) to quantify the navigation performance of a robot. The common metrics for navigation performance are shown in Table 4.

**TABLE 4:** Evaluation metrics for navigation performance.

Metric	Description
**Path Efficiency**	**The ratio between the distance between two waypoints and the length of the agent’s actual path between those points**
Publications: Qian et al. (2010a); Guzzi et al. (2013b); Kruse et al. (2013); Stein et al. (2016); Sebastian et al. (2017); Honig et al. (2018); Mavrogiannis et al. (2018); Neggers et al. (2018); Johnson and Kuipers (2019); Mavrogiannis et al. (2019); Vasconez et al. (2019); Ahmadi et al. (2020); Batista et al. (2020); Chadalavada et al. (2020); Liang et al. (2020); Hacinecipoglu et al. (2020); Zhang et al. (2021)	
**Success Rate**	**Ratio of successful trials**
Publications: Burgard et al. (1998); Jin et al. (2019); Nishimura and Yonetani (2020); Guzzi et al. (2013b); Tsai and Oh (2020); Liang et al. (2020); Chen et al. (2019b,a); Honig et al. (2018); Kamezaki et al. (2020); Qian et al. (2010b); Samsani and Muhammad (2021); Sprute et al. (2019); Yao et al. (2021)	

#### 4.1.1 Navigation Efficiency

We observed multiple measures of navigation efficiency in prior research, including path efficiency and relative throughput. Path efficiency is defined as the ratio of the distance of two waypoints to the length of the agent’s actual path between those points (Mavrogiannis et al., 2019). Relative throughput (Guzzi et al., 2013b) is defined as the ratio of the number of targets the agent can reach if it ignores all collision and social constraints to the number of targets an agent can reach in an actual simulation. Both metrics calculate a ratio of performance under an ideal condition to performance under the actual condition, indicating the influences of interactions—either with people or the environment—on navigation efficiency. Other metrics for assessing efficiency include average velocity and mean time to goal (Liang et al., 2020).

#### 4.1.2 Success Rate

In addition to the efficiency metrics discussed above, success rate is commonly used to quantify navigation performance in socially-aware navigation (Burgard et al., 1998; Guzzi et al., 2013b; Jin et al., 2019; Liang et al., 2020; Nishimura and Yonetani, 2020; Tsai and Oh, 2020). Success rate, or arrival rate, measures an agent’s ability to reach its goal. When reporting success rate, it is also common to disclose the number of collisions and timeouts (e.g., Chen C. et al., 2019; Nishimura and Yonetani, 2020); a navigation trial is considered “timed out” if the agent cannot reach its goal within a specified time limit.

It is worth noting that success rate is highly dependent upon the environmental context and does not differentiate the quality of navigation between successful trials. As a result, weighted success rate metrics have been proposed to consider aspects of navigation efficiency, such as path length and completion time, while assessing success rate. These weighted metrics are single, summary metrics that represent navigation performance and can be particularly useful in reinforcement learning, which is a popular method used in recent works on robot navigation (Anderson et al., 2018; Yokoyama et al., 2021).

### 4.2 Behavioral Naturalness

Metrics related to naturalness focus on low-level behavioral patterns, i.e., how human-like and smooth robot movements are; measures of human similarity and path smoothness are also commonly used in human trajectory prediction research (Rudenko et al., 2019). A summary of the metrics for behavioral naturalness are shown in Table 5.

**TABLE 5:** Evaluation metrics for naturalness.

Metric	Type		Description
	Similarity	Smoothness	
**Average Displacement Error (ADE)**	***✓***		**The average *L*_2_ distance between the predicted trajectory and the human data**
Publications: Pellegrini et al. (2009); Alahi et al. (2016); Bera et al. (2017); Gupta et al. (2018); Anderson et al. (2019); Manso et al. (2019); Rudenko et al. (2019); Kothari et al. (2020); Zou et al. (2020); Hacinecipoglu et al. (2020); Zhou et al. (2021)			
**Final Displacement Error (FDE)**	***✓***		**The distance between the final destination in the prediction and the human data at the same time step**
Publications: Rudenko et al. (2019); Anderson et al. (2019); Kothari et al. (2020); Gupta et al. (2018); Manso et al. (2019); Bera et al. (2017); Pellegrini et al. (2009); Alahi et al. (2016); Zou et al. (2020); Zhou et al. (2021)			
**Asymmetric Dynamic Time Warping**	***✓***		**A trajectory measure that doesn’t require both trajectories to have the same length**
Publications: Luber et al. (2012); Charalampous et al. (2016); Charalampous et al. (2017); Kostavelis et al. (2017); Avelino et al. (2021)			
**Velocity and Acceleration**		***✓***	**Basic dynamics measures**
Publications: Sisbot et al. (2005); Sisbot et al. (2007); Pandey and Alami (2010); Qian et al. (2010a); Qian et al. (2010b); Scandolo and Fraichard (2011); Kruse et al. (2012); Shiomi et al. (2014); Kollmitz et al. (2015); Kretzschmar et al. (2016); Truong et al. (2017); Truong and Ngo (2017); Claes and Tuyls (2018); Honig et al. (2018); Tail et al. (2018); Buchegger et al. (2019); Mavrogiannis et al. (2019); Papenmeier et al. (2019); Randhavane et al. (2019); Yoon et al. (2019); Zhong et al. (2019); Boldrer et al. (2020); Chadalavada et al. (2020); Fang et al. (2020); Hacinecipoglu et al. (2020); Ngo et al. (2020); Senft et al. (2020); Shiying et al. (2020); Gonon et al. (2021); Kivrak et al. (2021); Yao et al. (2021)			
**Path Irregularity**		***✓***	**The amount of unnecessary turning over the whole path**
Publications: Guzzi et al. (2013b); Mavrogiannis et al. (2018)			
**Topological Complexity**		***✓***	**Measures path entanglement to quantify encounters**
Publications: Kretzschmar et al. (2016); Mavrogiannis et al. (2018)			

#### 4.2.1 Movement Similarity

A common hypothesis in socially-aware navigation is that robots should possess navigational behaviors similar to humans’ (Luber et al., 2012; Kruse et al., 2013). As a result, many prior works focus on developing and evaluating methods of producing robot trajectories that resemble those of humans under similar conditions. These prior works use a variety of measures—including displacement errors, dynamic time warping distance, and Hausdorff distance—to directly assess similarities between trajectories and end states in navigational performances.

##### Displacement Errors

Displacement errors are a family of metrics typically utilized in evaluating how well a predicted trajectory matches human trajectory data or a trajectory derived from other baseline methods. These metrics are widely used in pedestrian trajectory prediction research (Anderson et al., 2019; Rudenko et al., 2019; Kothari et al., 2020); they are also applied as evaluation metrics to assess the similarities between trajectories produced by navigation algorithms and by humans (Bera et al., 2017; Gupta et al., 2018; Manso et al., 2019; Kothari et al., 2020).

• Average Displacement Error (ADE) is the average *L*_2_ distance between the predicted trajectory and the human data to which it is being compared. It was first used to evaluate trajectory similarity in socially-aware navigation by Pellegrini et al. (2009). As the nonlinear segments of a trajectory are where most of the social interactions between a robot and pedestrians occur (Alahi et al., 2016), ADE over these nonlinear portions provides a more specific metric for assessing human-robot navigational interaction.
• Final Displacement Error (FDE) is the distance between the final destination in the predicted trajectory and the human data at the same time step. It was proposed by Alahi et al. (2016) as a complement to ADE and nonlinear ADE.

Variations such as minimum, minimum over N, best-of-N, and top *n*% ADE and FDE are also employed by recent pedestrian trajectory prediction works (Anderson et al., 2019; Rudenko et al., 2019); these metrics distinguish the highest accuracy a prediction can achieve on human data, which is vital for trajectory prediction. However, accuracy is not a primary concern for socially-aware navigation research, which prioritizes learning general behavior patterns rather than generating exact matches of human trajectories; therefore, these variations are rarely applied to socially-aware navigation.

### Dynamic Time Warping Distance

While displacement metrics are useful in characterizing overall trajectory similarities, they are inadequate in delineating the similarities between motion behaviors at different speeds; mismatched moving speeds are especially relevant to robot navigation as mobile robots have diverse form factors, resulting in widely varying velocities when compared to humans. To address this limitation, Luber et al. (2012) took a different approach by focusing on the fact that trajectories are time-series data bearing resemblance to spoken language; they proposed a modified version of Dynamic Time Warping (Sakoe and Chiba, 1978)—an algorithm commonly used for matching spoken-word sequences at varying speeds—to transform one trajectory into another *via* time re-scaling. A dynamic time warping distance can then be calculated to compare trajectories produced by agents moving at different velocities.

#### 4.2.2 Smoothness

The smoothness of both the geometric path and the motion profile of a robot are two important contributing factors to natural, safe navigation (Mavrogiannis et al., 2017; Mavrogiannis et al., 2018; Mavrogiannis et al., 2019). Not only are irregular paths and jittery movements inefficient, but they can also discomfort nearby pedestrians (Fraichard, 2007); therefore, it is critical to evaluate the smoothness of a robot’s geometric path and motion profile in socially-aware navigation.

##### Path Irregularity

The smoothness of a trajectory can be characterized by the geometry of its path. For example, path irregularity (PI) (Guzzi et al., 2013b) measures the amount of unnecessary turning over the whole path a robot has traveled:

$$\text{PI}=\underset{\text{Path}}{\sum }\frac{\text{Robot Rotation}-\text{Min. Rotation Needed}}{\text{Unit Path Length}}$$ (1)

##### Topological Complexity

Prior research has also explored the use of the topological complexity index (Dynnikov and Wiest, 2007) to measure the level of entanglement in agents’ paths (Mavrogiannis et al., 2018; Mavrogiannis et al., 2019). Greater path entanglement means that the agents are more likely to encounter each other during navigation, thereby inevitably forcing movement impact. Moreover, trajectories with simpler topological entanglements have been shown to be more legible (Mavrogiannis et al., 2018).

##### Motion Velocity and Acceleration

Velocity and acceleration are typically used to characterize motion profiles; a robot navigating in human environments is expected to keep a maximum velocity that allows it to reach the target while still maintaining a smooth acceleration profile. As an example, Mavrogiannis et al. (2019) used acceleration per segment and average energy per segment, where energy is the integral of squared velocity, to capture change in their robot’s motion.

### 4.3 Human Discomfort

In this section, we present metrics used to measure human discomfort in socially-aware navigation. A summary of these metrics are shown in Table 6. We define discomfort as pedestrians’ level of annoyance, stress, or danger caused by the robot’s presence. Discomfort—either physical or psychological—is typically quantified by spatial models and subjective ratings (e.g., perceived safety).

**TABLE 6:** Evaluation metrics for human discomfort.

Metric	Type			Description	Proposed in
	Spatial	Groups	Safety		
**Personal space**	***✓***		***✓***	**Spatial compliance for individuals**	Hall (1966)
Publications: Pacchierotti et al. (2006); Kessler et al. (2011); Scandolo and Fraichard (2011); Torta et al. (2013); Shiomi et al. (2014); Tomari et al. (2014); Talebpour et al. (2015); Kollmitz et al. (2015); Lindner (2016); Luo and Huang (2016); Kodagoda et al. (2016); Truong and Ngo (2016); Truong et al. (2016); Truong and Ngo (2018); Forer et al. (2018); Vega-Magro et al. (2018); Fei et al. (2019); Rajamohan et al. (2019); Randhavane et al. (2019); Bachiller et al. (2021); Banisetty et al. (2021); Batista et al. (2020); Fang et al. (2020); Fuse and Tokumaru (2020); Ngo et al. (2020); Shiying et al. (2020); Vega et al. (2020); Neggers et al. (2021)					
**o/p/r-space**	***✓***	***✓***		**Spatial compliance for static groups**	Kendon (2010)
Publications: Kessler et al. (2011); Scandolo and Fraichard (2011); Torta et al. (2013); Shiomi et al. (2014); Tomari et al. (2014); Kollmitz et al. (2015); Talebpour et al. (2015); Batista et al. (2020); Kodagoda et al. (2016); Lindner (2016); Luo and Huang (2016); Truong et al. (2016); Truong and Ngo (2016); Fei et al. (2019); Rajamohan et al. (2019); Randhavane et al. (2019); Fang et al. (2020); Forer et al. (2018); Truong and Ngo (2018); Vega-Magro et al. (2018); Fuse and Tokumaru (2020); Ngo et al. (2020); Shiying et al. (2020); Vega et al. (2020); Bachiller et al. (2021); Banisetty et al. (2021); Neggers et al. (2021)					
**Social Force Model (SFM)**	***✓***		***✓***	**Measures social compliance by artificial forces**	Helbing and Molnár (1995)
Publications: Šochman and Hogg (2011); Anvari et al. (2015); Huang et al. (2018); Kivrak and Kose (2018); Yang et al. (2019); Katyal et al. (2021)					
**Extended social force model**	***✓***	***✓***	***✓***	**Adds support for social groups to SFM**	Moussaïd et al. (2010)
Publications: Yang et al. (2019); Katyal et al. (2021)					

#### 4.3.1 Spatial Models

##### Spatial Models for Individuals

The impact of a mobile robot’s navigational behavior on human comfort is difficult to quantify (Rios-martinez et al., 2013; Rios-Martinez et al., 2015; Kothari et al., 2020), as no universal “rules” are available for defining psychological comfort. Nevertheless, research suggests that the psychological comfort of humans is affected by interpersonal distance (Aiello, 1977; Baldassare, 1978; Greenberg et al., 1980). Proxemic theory (Hall, 1966) studies the function of the space an individual maintains for different social purposes in interpersonal interactions. According to Hall’s observation, an individual’s perceived personal space consists of several layers of concentric circles structured by their social functions, as presented in Table 7; however, according to Hall, most of his subjects were healthy business professionals from the northeastern seaboard of the United States. So these spaces may vary by culture and interaction context. Other representations—such as ovoids, concentric ellipses, and asymmetric shapes—have also been used to represent personal spaces and encode more complicated social rules (Rios-Martinez et al., 2015).

**TABLE 7:** Interpersonal spaces as defined by Hall (1966).

Space name	Range	Function
Intimate space	<.45m	Intimate interactions
Personal space	0.45–1.2 m	Friendly interactions
Social space	1.2*–*3.6 m	Buffer zone for coexistence
Public space	>3.6m	Public interactions

Among the four spaces laid out by Hall (1966), personal space is often used as the boundary of measuring perceived safety or social comfort—either as a no-go zone, where entering the space is counted as a violation of social comfort (Rios-martinez et al., 2013; Shiomi et al., 2014), or as the boundary of a potential function that assigns costs or penalties to robots entering that space (Amaoka et al., 2009; Truong and Ngo, 2018; Yang and Peters, 2019).

However, the circular representation of personal space as suggested by Hall (1966) is quite restrictive, as it does not adequately account for characteristics of human perception and motion. As a result, many works have explored different representations to consider face orientation (Amaoka et al., 2009; Truong and Ngo, 2016), approach pose (Truong and Ngo, 2018), and motion velocity (Helbing and Molnár, 1995; Truong and Ngo, 2016). Prior research has also leveraged empirical data from experiments to model complex and realistic uses of space (Gérin-Lajoie et al., 2008; Moussaïd et al., 2009). Most notably, the Social Force Model (SFM) (Helbing and Molnár, 1995), which has been widely used to simulate human navigation behavior in social contexts, represents the constraints of personal space as attractive or repulsive forces originating from each agent. Specifically, Eq. 2 describes how an agent *i*’s behavior is driven by a combination of forces:

• ${\vec{f_{i}}}^{des}$ : an attractive force that drives the agent to the desired goal.
• ${\vec{f_{i}}}^{obs}$ : the repulsive forces from obstacles.
• $\sum_{j}{\vec{f}}_{ij}^{social}$ : the sum of social repulsive forces from all other agents, *j*.

$$\frac{d\vec{v_{i}}}{dt}={\vec{f_{i}}}^{des}+{\vec{f_{i}}}^{obs}+\underset{j}{\sum }{\vec{f_{ij}}}^{social}$$ (2)

Although SFM was designed for simulating crowd behavior, it has inspired metrics seeking to quantify social comfort in socially-aware navigation. For instance, repulsive forces from obstacles and nearby agents can be used to quantify violations of social comfort and indicate “panic” behaviors in emergencies (Mehran et al., 2009). Truong and Ngo (2018) proposed the Social Individual Index (SII) to measure the physical and psychological safety of an individual. Similarly, Robicquet et al. (2016) proposed the Social Sensitivity index, which uses potential functions to model how agents interact; high social sensitivity indicates that an agent will tend to avoid other agents.

##### Spatial Models for Groups

The aforementioned measures consider agents individually, but we must also consider that people interact socially in group settings. Social groups can be categorized into static and dynamic groups; static groups are groups of people standing closely together and engaging in conversations as commonly seen at social events, whereas dynamic groups are groups of people walking together toward shared destinations.

Static, conversational groups can be modeled using *f-formation* (Kendon, 2010). F-formation is the spatial arrangement that group members maintain in order to respect their communal interaction space, where *o-space* is the innermost space shared by group members and reserved for in-group interactions; *p-space* surrounds the *o-space* and is the space in which members stand; and *r-space* is the outermost space separating the group from the outer world. Similar to individual discomfort, discomfort caused by a robot to a group may be measured by the robot’s invasion into either the *r-space* or the *o-space*, based on the f-formation of the group (Mead et al., 2011; Rios-martinez et al., 2013; Ferrer et al., 2017).

It is commonly observed that people walk together in dynamic social groups (Federici et al., 2012; Ge et al., 2012). In addition, individual people tend to stay away from social groups when walking (Efran and Cheyne, 1973; Knowles et al., 1976; Moussaïd et al., 2010). A mobile robot deployed in human environments must know how to behave around human groups by observing such inherent etiquette. To simulate dynamic social groups, Moussaïd et al. (2010) proposed the Extended Social Force Model (ESFM).^7^ As shown in Eq. 3, ESFM adds a new group term ${\vec{f}}_{i}^{group}$ that dictates intra-group dynamics to the original SFM. The group term, as defined by Eq. 4, is the summation of three forces: a cohesive force that defines attractions between group members ${\vec{f}}_{i}^{att}$ ; a repulsive force between group members ${\vec{f}}_{i}^{rep}$ ; and a gaze force ${\vec{f}}_{i}^{gaze}$ that aligns each agent with the center-of-mass of the social group, factoring in head orientation to simulate in-group social interactions.

$$\frac{d\vec{v_{i}}}{dt}={\vec{f_{i}}}^{des}+{\vec{f_{i}}}^{obs}+\underset{j}{\sum }{\vec{f_{ij}}}^{social}+{\vec{f_{i}}}^{group}$$ (3)

$${\vec{f_{i}}}^{group}={\vec{f_{i}}}^{att}+{\vec{f_{i}}}^{reb}+{\vec{f_{i}}}^{gaze}$$ (4)

Similar to spatial models for individuals, spatial models for groups can be used to approximate discomfort in group interactions. As an example, to evaluate a robot’s social compliance as a group member when accompanying humans, Ferrer et al. (2017) proposed a quantitative metric based on the robot’s position in relation to the human members, accounting for whether or not the robot was in the field of view of the human members and the distances between group members.

#### 4.3.2 Physical Safety

Safety is the preeminent concern in socially-aware navigation. At the most basic level, navigational safety amounts to collision avoidance: a mobile robot should not have any physical contact—intentional or otherwise—with any human being. Metrics based on collision count or violation count are commonly used in simulated environments and in some robot-only experiments. For example, Liu L. et al. (2020) used the number of collisions with agents within and without the test agent’s field of view, along with success rate, as the main evaluation metrics in conducting their assessment of their deep reinforcement learning based navigation algorithm in simulation. Guzzi et al. (2013b) used small-scale robots in physical experiments, allowing them to use collision count as one of their main metrics in evaluating the impact of safety margin size.

While they are arguably the most straightforward methods of measuring navigational safety violations, collision and violation counts are neither practical nor ethical to use in real-world experiments and deployments involving humans, as collisions present potential harm to the participants. Consequently, safety violations should be approximated by invasions of defined safety zones. A safety zone is typically derived from the proxemics theory proposed by Hall (1966), wherein the personal space—ranging from 0.45 to 1.2 m in Western culture—is used to measure how well a mobile robot maintains the physical safety of nearby human pedestrians (e.g., Vega et al., 2019b). Variations on safety zones are frequently used in prior works; for example, the Collision Index (CI) (Truong and Ngo, 2016), or Social Individual Index (SII) (Truong and Ngo, 2018), is a distance-based metric for capturing the violation of personal space. The index is specified in Eq. 5, where $\left(x_{i}^{p},y_{i}^{p}\right)$ is the position of the *i*th pedestrian *p*_*i*_, (*x* ^*r*^, *y* ^*r*^) is the position of the robot, and $\sigma_{0}^{px}$ and $\sigma_{0}^{py}$ are the standard deviations of the personal space, empirically set to the value of 0.28:

$$\text{CI}=\underset{i=1:N}{\max}exp\left(-\left(\frac{x^{r}-x_{i}^{p}}{\sqrt{2}\sigma_{0}^{px}}+\frac{y^{r}-y_{i}^{p}}{\sqrt{2}\sigma_{0}^{py}}\right)\right)$$ (5)

In the original definition of the index (Truong and Ngo, 2016), the standard deviations are the same for both directions ( $\sigma_{0}^{px}=\sigma_{0}^{py}$ ), thus assuming that personal space is a perfect circle. However, as we discussed earlier, additional representations of personal space have been proposed to capture nuanced social rules, cultural influences, and specific situations (Rios-Martinez et al., 2015); therefore, this index may be adapted to account for different cultures, types of relationships, and interaction contexts by modifying the standard deviations. As another example of custom safety zones, Jin et al. (2019) defined the ego-safety zone as a circular space around an agent, analogous to the personal space, and the social-safety zone as a rectangular region stretching along an agent’s current moving direction.

#### 4.3.3 Psychological Safety

In addition to preserving physical safety, it is important to evaluate the effects of socially-aware navigation on psychological safety. Preserving psychological safety, or sometimes referred to as perceived safety, involves ensuring a stress-free and comfortable interaction (Lasota et al., 2017). Although they may not physically endanger a person, a mobile robot’s navigational behaviors (e.g., how they approach and pass a person) may yet induce feelings of discomfort or stress (Butler and Agah, 2001). Consider a situation in which a mobile robot moves rapidly toward a person and only changes its moving direction right before the imminent collision; while the robot does not make direct physical contact with the person, its navigational behavior is still likely to cause them significant stress.

A common method of assessing people’s perceived psychological safety is through questionnaires. Butler and Agah (2001) asked participants to rate their comfort from 1 to 5 (with 1 being very uncomfortable and 5 being very comfortable) under different experimental conditions, including varying robot speed, distance from the human subject, and approach patterns. Similarly, Shiomi et al. (2014) used a survey to assess people’s experiences interacting with a deployed mobile robot during a field study; specifically, the inquiry focused on three aspects: whether the interaction was free from obstruction, whether the person could maintain their preferred velocity in the presence of the robot, and their overall impression of the encounter.

Several established questionnaires designed for social robotics research already include questions regarding psychological safety. For example, the Godspeed questionnaire (Bartneck et al., 2008) has a sub-scale, perceived safety, comprised of questions related to subjects’ relaxed/anxious, calm/agitated, and surprised/quiescent emotional states. The Robotic Social Attributes Scale (RoSAS) (Carpinella et al., 2017), based on the Godspeed questionnaire, measures people’s perception and judgement of the robots’ social attributes, including warmth, competence, and discomfort. The BEHAVE-II instrument (Joosse et al., 2013) includes a set of behavioral metrics that measure human responses to a robot’s behavior; some of the metrics were specifically designed to gauge the discomfort caused by a robot’s approach behavior (e.g., a person’s step direction and step distance when a robot intrudes upon their personal space). Joosse et al. (2021) used this instrument to measure people’s responses to and tolerance of personal space invasion when being approached by agents at varying speeds.

### 4.4 Sociability

We define sociability as a robot’s conformity to complex, often nuanced, social conventions in its navigational behavior. Previously, we have described various metrics used to measure motion-level social conventions, such as approach velocity, approach pose, invasion of personal space, or passing on the dominant side (e.g., Truong and Ngo, 2016; Guzzi et al., 2013b; Yang and Peters, 2019; Pacchierotti et al., 2006). However, there exist more complex social norms around navigation-based interactions, such as elevator etiquette, waiting in a queue, asking permission to pass, and observing right-of-way at four-way intersections. A robot may move in a natural and appropriate manner that does not cause discomfort, but still violates expected, high-level social norms. For example, a robot may enter an elevator full of people in a perfectly smooth and natural fashion without first letting anyone inside leave; while the robot does not exhibit any unnaturalness or cause discomfort by violating motion-level social conventions, it breaks higher-level social norms that most people expect when riding an elevator. Measuring these high-level social norms would allow for a more holistic understanding of the impact of robot presence on humans; however, measuring sociability remains largely difficult and is considered one of the key challenges in the field of socially-aware navigation (Mavrogiannis et al., 2021).

The Perceived Social Intelligence (PSI) scales proposed by Barchard et al. (2018); Barchard et al. (2020) evaluate 20 aspects of robotic social intelligence. For instance, the Social Competence (SOC) scale consists of four items: 1) social competence, 2) social awareness, 3) social insensitivity (reversed), and 4) strong social skills. PSI scales have been used in previous evaluations of socially-aware navigation (e.g., Barchard et al., 2020); recently, Banisetty and Williams (2021) used the perceived safety scale from the Godspeed questionnaire in conjunction with PSI to evaluate how a robot’s spatial motions may communicate social norms during a pandemic via an online study. Additionally, it has been determined that robots using socially-aware navigation planners are perceived to be more socially intelligent as measured by PSI than those using traditional navigation planners (Honour et al., 2021).

In addition to using validated scales, prior research has employed custom questions relevant to specific evaluation contexts to gauge people’s perceptions of robot sociability. For example, Vega et al. (2019a) used three questions—*Is the robot’s behavior socially appropriate?*; *Is the robot’s behavior friendly?*; and *Does the robot understand the social context and the interaction?*—to evaluate how a mobile robot may interact with people to ask for permission to pass when they block its path. All in all, how best to measure sociability remains unresolved, as opposed to the consensus on metrics for evaluating navigation performance and trajectory similarity.

## 5 Discussion

In this paper, we review the evaluation protocols—focusing on evaluation methods, scenarios, datasets, and metrics—most commonly used in socially-aware robot navigation with the goal of facilitating further progress in this field, which currently lacks principled frameworks for development and evaluation. Prevalent evaluation methods include simulation experiments followed by experimental demonstration, as well as laboratory and field studies. Controlled experiments, either in simulation or in the physical world, typically focus on a set of primitive scenarios such as passing, crossing, and approaching. Datasets of human movements and trajectories are regularly utilized in developing and evaluating socially-aware navigation policies. Prior works have also explored a range of objective, subjective, and behavioral measures to evaluate navigation performance, naturalness of movement, physical and psychological safety, and sociability. Below, we discuss limitations of the existing evaluation protocols and open problems to solve in future research.

### 5.1 Limitations of Existing Evaluation Protocols

#### 5.1.1 Evaluation Methods, Scenarios, and Datasets

Recent works on socially-aware navigation rely heavily on datasets and simulation experiments for evaluation (Mavrogiannis et al., 2021); this trend has been accelerated by advances in reinforcement learning and data-driven approaches in general (e.g., Luber et al., 2012; Zhou et al., 2012; Alahi et al., 2014; Alahi et al., 2016; Kretzschmar et al., 2016; Park et al., 2016). However, this type of evaluation makes strong assumptions about human and robot behaviors. For example, in simulation experiments, researchers typically rely on pedestrian behavior models such as Optimal Reciprocal Collision Avoidance (ORCA) (Van Den Berg et al., 2011) (e.g., Chen et al., 2017b; Daza et al., 2021) and the Social Force Model (SFM) (Helbing and Molnár, 1995) (e.g., Katyal et al., 2021). Reciprocal behavior models such as ORCA impose the assumption that each agent is fully aware of its surroundings and the position and velocity of the other agents; this assumption of omniscience does not hold true for a real robot or person (Fraichard and Levesy, 2020). Moreover, agents trained using ORCA and SFM behave much differently than real-life agents (Mavrogiannis et al., 2021) and there exist a multitude of SFM variations (e.g., Moussaïd et al., 2009; Anvari et al., 2015; Truong and Ngo, 2017; Huang et al., 2018; Yang and Peters, 2019); therefore, it is important to ensure comparable settings for training and evaluation when comparing algorithms in simulation experiments.

To add to this concern of agent behavior assumptions, the simulators used in virtual social navigation experiments have their own limitations. While 2D simulators such as Stage (Gerkey et al., 2003) and CrowdNav (Chen C. et al., 2019) are lightweight and easy to extend, they oversimplify and abstract, rendering their results difficult to apply to the real world. Recently, several high-fidelity, photorealistic simulation environments were developed for indoor navigation, such as Matterport 3D (Chang et al., 2017) and Gibson (Xia et al., 2018). These environments offer improved simulations closer to real-world settings; however, generating realistic, grounded human social behaviors in high-fidelity simulation environments is still challenging.

Simulation experiments typically leverage datasets and metrics that quantify performance and similarity as described in Section 4.2.1. This reliance on datasets and quantitative metrics assumes that the human behaviors recorded in those datasets represent the optimal behaviors for a robot—despite robots possessing dynamics and dimensions largely dissimilar to humans; at best, it is highly debatable whether an exact copy of human trajectories is socially acceptable for all robots. Finally, as described in Section 3.1, simulation experiments are commonly followed by demonstrations with physical robots in a real-world setting; while appropriate for proofs-of-concept, these demonstrations are mainly illustrative and lack statistical rigorousness.

In contrast, laboratory studies allow for controlled experiments with statistical precision. However, such experiments are often simplistic and designed for specific navigational interactions (Table 2) in certain settings (e.g., passing interactions in a hallway). Moreover, it is important to note that interaction scenarios are usually evaluated out of context. Take the crossing scenario as an example; although crossing is largely evaluated in an open setting (e.g., circle crossing), people may exhibit very different crossing behaviors in real life, as shaped by their individual objectives, other pedestrians, and the environment (e.g., in an open square or an art gallery). Furthermore, laboratory studies typically rely on convenience sampling for participant recruitment (e.g., college students and local residents), resulting in findings that may have limited generalization to a broader population.

Field studies are arguably the most challenging evaluation method to execute; they require robots to operate robustly and safely in unstructured human environments and naturally involve emergent, unprescribed human-robot interactions. While challenging and costly, field studies can provide rich, and sometimes unexpected, insights that simulation and laboratory studies cannot offer (Section 3.1.4).

Going forward, we predict an increased need for bridging algorithmic innovations in simulation and autonomous, real-world interactions. Deploying robots for human interaction, either in the field or in laboratory settings, will help us better understand the true limitations of robotics technology and how people experience and interact with it. We strongly advocate for more laboratory and field studies to productively advance socially-aware robot navigation and develop useful, functional mobile robots.

#### 5.1.2 Evaluation Metrics

##### Navigation Performance

Socially-aware robot navigation shares many performance metrics with general robot navigation. Conventional performance metrics, such as efficiency and success rate, are commonly reported in the literature of socially-aware robot navigation. For example, path efficiency is the ratio of the optimal path’s length to that of the actual path and is used to measure path disturbance to agents (either the robot or human pedestrians), while success rate measures an agent’s ability to reach its goal. Though not typically used in evaluating socially-aware navigation, we believe metrics that account for both path efficiency and success rate, such as Success weighted by Path Length (SPL) (Anderson et al., 2018), Success weighted by Number of Actions (SNA) (Chen et al., 2021), and Success weighted by Completion Time (SCT) (Yokoyama et al., 2021), are useful metrics to compare navigation policies. However, these metrics should only be used for comparisons in the same setting, as different settings have different optimalities. All in all, these metrics attempt to sum up navigation trials into singular values; while such abstraction is useful for systematic comparison, it makes the assessment of fine-grained trajectory quality more difficult. To answer questions like what caused a particular defect in efficiency, researchers typically visualize trajectories for more qualitative analysis. However, it is worth noting that the most socially acceptable navigational behaviors are not necessarily efficiency- or performance-oriented.

##### Naturalness

A common method of measuring naturalness is quantifying the similarity between the robot’s or the predicted trajectory and those observed in human data. Average Displacement Error (ADE) and Final Displacement Error (FDE) are conventional metrics for quantifying trajectory differences. Variations of displacement- or distance-based metrics may be employed to highlight certain aspects of navigation; for instance, ADE over the nonlinear portions of a trajectory may capture the effects of navigational interactions (e.g., passing and crossing). These types of metrics are typically used in benchmarking navigation algorithms against provided datasets in simulation experiments. While allowing for reproducible and systematic development and evaluation, this dataset-oriented evaluation protocol has several limitations. First, human navigational behaviors and trajectories are context-dependent. The recorded human behaviors in a dataset are specific to the scenario in which the data was collected; moreover, most datasets only include a limited number of scenarios. Therefore, the generalizability of the evaluated algorithms to different contexts is not adequately captured by these metrics. Second, robots and humans afford distinct navigational behaviors and expectations. At the physical level, robots are quite dissimilar to humans and therefore afford different navigational behaviors, such as moving speed. At the social level, it has been revealed that people exhibit different social expectations toward robots than humans; for instance, empirical data suggests that people are willing to let robots get closer to them than they let fellow humans (Joosse et al., 2021). Finally, the majority of existing datasets are limited to 2D trajectories and neglect the fact that navigational behaviors are multimodal in nature. Such limitations necessitate the inclusion of additional metrics to cover aspects of naturalness like sociability and interaction quality.

Instead of using recorded human trajectories as a gold standard for assessing naturalness, several context-independent metrics have been utilized to measure movement smoothness, which is regarded as an important indicator of naturalness. These metrics usually consider velocity and acceleration profiles and path irregularity, which captures the number of unnecessary turns in a path. However, appropriate interpretation of the results from these metrics requires reference points (e.g., is a path irregularity value of 0.72 “good?”) that are difficult to obtain and may depend on various factors such as environmental context and culture.

##### Discomfort

Discomfort is another key dimension in which socially-aware robot navigation is evaluated; it can be characterized generally by physical and psychological safety. To approximate discomfort, prior works have relied upon spatial models including Hall (1966) theory on proxemics and personal space, *f-formation* for groups (Kendon, 2010), the Social Force Model (SFM) (Helbing and Molnár, 1995), and the Extended Social Force Model (ESFM) (Moussaïd et al., 2009). These models are particularly relevant to and useful in evaluating mobile navigation and spatial relationships; specifically, they have been adapted to define safety zones and identify abnormal behaviors (e.g., invading personal space) that may cause discomfort. For instance, prior research has used the Social Individual Index (SII), a numerical metric derived from spatial models, along with empirically determined thresholds to gauge psychological safety (Truong and Ngo, 2017). However, spatial model-based metrics are limited in several ways. First, all agents are assumed to be identical (e.g., possessing the same personal space and social forces), neglecting individual differences observed in the real world; for instance, how people distance themselves from others depends upon personal relationships, individual characteristics, interaction contexts, and cultural norms. Second, common spatial models do not have sufficient granularity to represent environmental contexts. As an example, in SFM, repulsive forces from the environment are all treated the same; however, people move and interact differently in different contexts, and are therefore likely to have varying levels of discomfort tolerance in response to robot navigational behaviors. Third, it is difficult to encode high-level social norms (e.g., sociability) into these spatial models. Altogether, spacial model-based metrics are limited in their ability to represent, simulate, and quantify complex, nuanced social behaviors that humans expect and exhibit in navigation.

In addition to using the aforementioned metrics, discomfort may be measured by self-report ratings [e.g., the perceived safety subscale from the Godspeed questionnaire (Bartneck et al., 2008)] and behavioral indices [e.g., the BEHAVE-II instrument (Joosse et al., 2013)]. These measures are effective in revealing people’s subjective experiences and genuine behavioral responses, which may not be accurately represented by objective metrics derived from spatial models. It is worth noting that these subjective and behavioral measures are collected after experiment completion and are consequently unsuitable for learning or adapting robot behavior in real time; however, some of the behavioral measures (e.g., step distance, facial expressions, and eye gaze) from BEHAVE-II may be calculated using computer vision techniques and therefore have the potential to be utilized in real-time behavioral adaptation.

##### Sociability

Sociability is a complex construct that characterizes a robot’s conformity to high-level social conventions, which are conditioned on varying factors such as culture, interaction and environmental contexts, and individual characteristics (e.g., gender); as a result, there are no predetermined sets of high-level social conventions. Therefore, research thus far has explored social conventions that are by and large cherry-picked by the researchers themselves. For example, Pacchierotti et al. (2006) defined a set of social rules for hallway interactions, suggesting that a robot should 1) signal its intention by proactively moving to the right; 2) stay as far away from humans as the width of the hallway allows; and 3) wait until a person completely passes by before resuming normal navigation in order to avoid causing discomfort. Salek Shahrezaie et al. (2021) emphasized that social rules differ based on environmental contexts; for instance, a robot will need to behave differently in galleries, hallways, and around vending machines. The wide range of influencing factors on sociability makes it challenging to adopt a uniform evaluation standard or set of metrics. As a consequence, most prior works adopted an ad hoc approach, using custom questions to assess sociability (e.g., Vega et al., 2019a). More recently, Perceived Social Intelligence (PSI) scales (Barchard et al., 2020) offer an initial point for benchmarking the subjective construct of sociability. In order to productively advance socially-aware navigation, however, further research is required to develop comprehensive instruments specifically designed to measure sociability and higher-level social skills in the context of navigational interactions.

### 5.2 Open Problems and Opportunities

#### 5.2.1 Diverse, Dynamic Human Models and Long-Term Effects

As discussed in Section 5.1.1, there are several limitations to simulation-based evaluation, the most notable of which being homogeneity—all agents are driven by a static behavior engine—and omniscience—all agents have full awareness of their surroundings (Fraichard and Levesy, 2020); these assumptions are a result of the oversimplification and abstraction built into simulators. Moreover, most spatial models for crowd behavior and proxemics are derived from population data; consequently, the experiments and simulations using them often do not support a sufficiently diverse representation of different groups of people (Hurtado et al., 2021). Indeed, humans are naturally diverse and their behaviors and expectations change over time and according to complex factors like individual traits, cultures, and contexts. For example, abundant empirical evidence has demonstrated how age (e.g., Nomura et al., 2009; Flandorfer, 2012), personality (e.g., Walters et al., 2005; Robert, 2018), gender (e.g., Flandorfer, 2012; Strait et al., 2015), and cultural (e.g., Lim et al., 2020) differences may affect people’s perceptions of and interactions with robots. Moreover, similar to how people gradually change their behaviors (e.g., standing closer when talking to each other) to reflect developments in a relationship (Altman and Taylor, 1973), robots must also evolve their behaviors—as opposed to exhibiting behaviors uniformly over time—to match their relationships and promote rapport with users. Not only must we develop behavior models to account for gradual changes in relationships, but we must conduct more longitudinal studies to explore how people’s experiences with, perceptions of, and behaviors toward robots change over long periods of time. Buchner et al. (2013) demonstrated that a person’s experience with a collaborative robot clearly changes over the course of a year; will we see similar effects in navigational human-robot interactions? Ultimately, we have three recommendations for future research:

• Enrich pedestrian models: Although there are limitations to simulation-based approaches to socially-aware navigation, these approaches allow for rapid development and systematic benchmarking and are particularly useful for early-stage validation. However, future simulation-based research must augment pedestrian models to account for human diversity; this may be achieved by including variables to represent the influencing factors we previously discussed and by introducing parameters to regulate said variables over time and according to interaction contexts.
• Examine longitudinal effects: Our understanding of the longitudinal effects of navigational human-robot interactions is fairly limited, yet such knowledge is critical in developing and integrating mobile robots into real-life environments with the goal of interacting with and assisting people in their daily lives. As the field of socially-aware robot navigation continues to evolve, research efforts should increasingly concentrate on conducting longitudinal field studies.
• Measure and report individual characteristics: As previously mentioned, many characteristics and factors demonstrably influence general human-robot interaction. To collectively advance our understanding of navigational human-robot interaction, we encourage future works to collect and report data on individual characteristics (e.g., age, personality, gender, and culture) and how they relate to the metrics of socially-aware navigation.

#### 5.2.2 Evaluating Mobile Robots of Different Forms

In this paper, we focus on the evaluation of socially-aware navigation in typical mobile robots that move around and interact with people in human environments, such as indoor or outdoor delivery robots. However, mobile robots can take many forms, interactions with humans can happen in different settings (e.g., where people are “on” or “inside” the robot), and human environments can include larger-scale infrastructures such as roads and highways. In particular, our review does not address two notable classes of “robot”: robotic wheelchairs and autonomous vehicles. While these two categories share various characteristics in terms of socially-aware navigation, they necessitate additional evaluation considerations and methods.

Similar to traditional mobile robots, robotic wheelchairs must consider the people around them when moving through human environments (e.g., Kretzschmar et al., 2016); as such, various evaluation considerations and metrics discussed in this paper may be adapted for this category of “robot.” However, robotic wheelchairs must also take into account additional considerations for their direct users; for instance, Morales et al. (2015) explored ways of including human factors (e.g., user visibility of the environment) when planning paths for a robotic wheelchair and evaluated how comfortable users felt during the ride. In support of greater accessibility and equity, more research is needed to investigate developing and evaluating methods that enable people who are robotic wheelchair-bound to engage in social interactions with individuals or groups of people (e.g., joining or following a social group) (e.g., Escobedo et al., 2014); as such, robotic wheelchairs should consider both users’ and surrounding pedestrians’ social signals (e.g., intent to interact). The navigation evaluation should also include behavioral indices that capture such nuanced social dynamics. Moreover, as robotic wheelchair users have varying physical disabilities, the development and evaluation of socially-aware navigation capabilities for robotic wheelchairs must pay closer attention to individual needs. Accordingly, custom metrics may be more appropriate for evaluation, as opposed to relying upon a rigid set of standardized evaluation protocols. Detailed reporting of user characteristics and specific needs would help contextualize evaluation results.

Autonomous vehicles (AVs) are up-and-coming “mobile robots” that interact with humans, including the “driver,” pedestrians, and other motorists on the road. Like traditional delivery robots, AVs must drive in a safe and predictable manner, but beyond excellent safety protocols and autonomous capabilities, AVs also require critical social awareness; social interactions underlie all pedestrian-vehicle interactions (Rasouli and Tsotsos, 2020) and even AV-AV interactions are considered social coordination events (Schwarting et al., 2019). Similar to evaluating robotic wheelchair applications, the evaluation of AV technology must consider a range of stakeholders, including pedestrians (e.g., Randhavane et al., 2019; Camara et al., 2021), bicyclists (e.g., Rahman et al., 2021), and other drivers (e.g., Schwarting et al., 2019). However, AV evaluation poses additional challenges (e.g., legal regulation for high-stake, life-critical applications) and has different considerations and norms (e.g., following traffic rules). To mitigate safety concerns, recent research has leveraged modern immersive technology such as virtual reality (VR) (e.g., Goedicke et al., 2018; Mahadevan et al., 2019; Camara et al., 2021) when evaluating socially-aware AVs; for instance, Camara et al. (2021) did their user study in a virtual reality setting to evaluate pedestrians’ behavior when crossing road with vehicles present. Similar to the evaluation for mobile robots, it is very important to measure the subjective perception of pedestrian-vehicle interactions (Mahadevan et al., 2019) and consider unique spatial interactions in AV applications.

To conclude, we expect to see more autonomous mobile technologies coexisting with people in their daily lives. While these technologies—ranging from mobile service robots and robotic wheelchairs to autonomous vehicles—may have domain-specific considerations for their development and evaluation, social awareness will be vital to the successful adoption of these technologies by the general population.

## 6 Conclusion

As the field of socially-aware navigation continues to evolve, it is vital to cultivate principled frameworks for the development and evaluation of mobile robots that aim to navigate in human environments in an efficient, safe, and socially acceptable manner. In this paper, we review the evaluation protocols commonly used in socially-aware robot navigation as an effort toward developing a principled evaluation framework. Our review highlights the advantages and disadvantages of different evaluation methods and metrics; in particular, while simulation experiments allow for agile development and systematic comparisons, laboratory and field studies can offer valuable insights into navigational human-robot interactions. Moreover, objective, subjective, and behavioral metrics used together offer a more comprehensive view of robot navigation performance and user experience than individual sets of metrics alone. By reviewing evaluation protocols for socially-aware robot navigation, this paper contributes to the broader vision of successful integration of socially-aware mobile technologies into our daily lives.

## Footnotes

1 Japan Post Co. piloted their mail delivery robot in Tokyo in October 2020.

2 FedEx is currently developing the SameDay Bot for package delivery.

3 Domino’s launched delivery robots in Houston, TX, United States in April 2021.

4
https://www.cyberbotics.com

5
https://www.aicrowd.com/challenges/trajnet-a-trajectory-forecasting-challenge

6
http://svl.stanford.edu/igibson/challenge.html

7 Our implementation of ESFM—https://github.com/yuxiang-gao/PySocialForce
